# Birthplace Effect in Soccer: A Systematic Review

**DOI:** 10.5114/jhk/186935

**Published:** 2024-07-17

**Authors:** Lander Hernández-Simal, Julio Calleja-González, Alberto Lorenzo Calvo, Maite Aurrekoetxea-Casaus

**Affiliations:** 1 Faculty of Education and Sport, University of Deusto, Bilbao, Spain.; 2 Faculty of Education and Sport, University of Basque Country (UPV/EHU), Vitoria, Spain.; 3 Faculty of Kinesiology, University of Zagreb, Zagreb, Croatia.; 4 Faculty of Education and Sport, Universidad Politécnica de Madrid, Madrid, Spain.; 5 Faculty of Social Sciences and Humanities, University of Deusto, Bilbao, Spain.

**Keywords:** talent development, talent hotspots, soccer, environment

## Abstract

Birthplace is a contextual variable that influences the talent development process of soccer players. The main objective of this systematic review was to compile the scientific literature on the effect of birthplace in soccer. This is the first systematic review to investigate how this contextual factor relates to talent development in soccer. Using the PRISMA methodology, the analysis of the 14 articles that were part of the final review concluded with three main discursive themes: the place of birth and population size, composition of the birthplace and socio-demographic characteristics of the population, and the place of birth and the location of high performance sport centres. This review shows that there are several underlying elements for understanding the effect of the place of birth on the development of talent in soccer. The combination of population density and the level of facilities in the place where the player was born are key in the development of a soccer player. It is not only about the place as a space, but also about what that space means for the development of the player's soccer skills. The findings not only highlight the characteristics of the areas, in terms of density and equipment supply, identified as talent hotspots, but also provide an opportunity to critically reflect on current practices of talent identification and development in soccer.

## Introduction

Historically, researchers have been interested in understanding the factors that affect the identification and development of the best sports performance among elite athletes (Fuhr et al., 2022; Spies et al., 2022; Xiang et al., 2022). Clearly, such processes must be complex, given that elite athletes are the product of numerous factors that influence their growth (Baker et al., 2018; Chmura et al., 2022; Verbeek et al., 2023). In this regard, Baker and Horton (2004) proposed dividing the influential aspects of players’ development into primary and secondary categories. Those considered primary directly affect the athlete’s performance (e.g., genetic or psychological dispositions and training patterns), while the secondary factors have an indirect, albeit still important, influence. Among these are contextual factors (Leite et al., 2021), the first of which is the athlete’s birthplace (Côté et al., 2006). Together with the relative age effect (Lemoyne et al., 2023; Pelletier and Lemoyne, 2022), these factors comprise the most studied environmental factors in the recent scientific literature (Leite et al., 2021).

Carlson (1988) and Curtis and Birch (1983) were the first to explore how geographical variables could influence the development of sporting talent, success in athletic training, and general athletic excellence. This phenomenon extends beyond simple geography, as it involves the complexity of socio-economic, cultural, infrastructural, and other factors that affect both access and opportunities to participate in sports, thus profoundly influencing the processes of talent identification and development (Hancock et al., 2022). In parallel to the effect of the birthplace, it is important to note that this phenomenon has historically been analysed in a variety of countries and sport modalities perhaps even as many as the contexts in which athletes emerge. Studies have crossed international borders and covered a wide range of disciplines, thereby reflecting the universality of the issue and its relevance to understanding the development of sporting talent worldwide. From ice hockey in North America (Baker and Logan, 2007; Côté et al., 2006) to cricket in the United Kingdom (Low et al., 2015), researchers have sought to understand how the geographic and demographic characteristics of a region contribute to sporting success (Bruner et al., 2011; MacDonald et al., 2009a; Pennell et al., 2017; Wattie et al., 2018) and, specifically, the role that birthplace plays in this success (Hancock et al., 2022).

Since the 1980s, a new body of research has emerged that dates back to previous decades, when the basic foundations for understanding the phenomenon of the birthplace in sport were laid. This trajectory has motivated us to conduct a historical review that not only illuminates the evolution of this field of study, but also sets the stage for current explorations. In this context, the “birthplace effect” is defined as the impact that the place of origin where the athlete was born and spends his/her early years of development has on his/her potential for access to and success in sport (Baker et al., 2009).

Existing research emphasises the need to understand how the birthplace can provide advantages for the development of sporting talent. These advantages are not only geographic coincidences, but also the result of policies and practices that can influence the distribution of certain opportunities (Farah et al., 2019; Hernández-Simal et al., 2024a).

Therefore, the birthplace effect is closely related to the early stages of athletic development and may be a determining factor in reaching higher levels of sporting proficiency (De Carvalho et al., 2018).

One of the most widely used mechanisms to explain the extent to which the birthplace has an effect on the development of sporting talent has been population size (Baker et al., 2009; Ishigami, 2016; MacDonald et al., 2009b; Rossing et al., 2016). Thus, several studies support the idea that it is more beneficial for young athletes to grow up in small- or medium-sized cities than in large cities (Nieuwstadt et al., 2021). Although large cities may offer children and young people better conditions, such as well-designed and equipped sport facilities and better coaching guidance, it seems that sports programmes in large cities are essentially structured around the assumption that young athletes lack the space and time to participate and practice extensively (Waalkes, 2017). In contrast, the physical environments of smaller cities or towns allow for educational and social benefits that cannot be obtained in large cities (Baker and Logan, 2007). More quantitatively, Smith and Weir (2020) concluded that athletes born in smaller cities (< 500,000 inhabitants) were more likely to play in professional leagues compared to athletes born in larger cities (> 500,000 inhabitants).

Along with population size, studies have focused on another variable closely linked to size, i.e., population density. Hancock et al. (2018) found that the population density of the birthplace was significantly lower for players on teams in the premier league (the highest level of soccer competition in the country) than for players on teams in the third league (a lower level of competition). The role of population density has been reflected in the performance variations of both different countries and sports, and at both the individual and collective levels (Côté et al., 2006), although authors such as Ishigami (2016) have argued that population density does not influence the probability of becoming an elite professional athlete.

In addition to size and density (Hancock et al., 2018), there are many other environmental features that characterise the community in which a sport is played and that the scientific literature highlights as influential in the relationship between the effect of the birthplace and the development of sporting talent. For example, access to facilities (Rossing et al., 2018) and open spaces or proximity to sports clubs and/or organisations can influence the level of activity in which young people engage (Kelly et al., 2021). Other characteristics analysed in relation to the birthplace include the socio-economic and cultural status of territorial communities (Federico et al., 2009; Hernández-Simal et al., 2024b).

Despite the emergence of studies addressing the effects of the birthplace and the resulting complexities of talent management, the diversity of variables considered makes it difficult to predict the actual future performance of athletes (Williams et al., 2020). Debate thus persists on whether the focus of this effect should be on the actual birthplace or the place of development, as athletes are often not born where they develop (Hancock et al., 2022). In an attempt to define this phenomenon more precisely, researchers have termed it the “early developmental place effect” to more accurately highlight the pivotal role that these different places occupy in young athletes’ developmental years (Rossing et al., 2018).

Research findings remain inconclusive, and there are significant contradictions across studies caused by the territorial origins of the studied athletes (Asso, 2021); specifically, differences between sports and countries in terms of population size and the quality of life have influenced the results (Hancock et al., 2018). These inconsistencies have led researchers to conclude that studies should examine other variables (Farah et al., 2019) that could contribute to an ideal environment for talent development in sports, such as proximity to the sport infrastructure (Rossing et al., 2018) or the quality of life indices (Teoldo and Cardoso, 2021).

Given this scenario, this research was planned as a systematic review to determine the extent to which the birthplace has been shown to influence the development of talent in sports performance. More specifically, the aim was to compile all published scientific literature related to the birthplace in soccer, since to the authors’ knowledge, no study has yet compiled the existing literature related to the effect of the birthplace on soccer performance.

## Methods

### Bibliographic Search Strategies

This systematic review was conducted following the Preferred Reporting Items for Systematic Review and Meta-Analyses (PRISMA^®^) guidelines (Page et al., 2021) and the Patient, Intervention, Comparison, Outcome (PICOS) model for the definition of inclusion criteria (Methley et al., 2014). The PICOS model is shown in Table 1.

**Table 1:** PICOS model for inclusion criteria definition

P (Population)	‘soccer players’
I (Intervention)	‘birthplace effect’
C (Comparators)	‘not applicable’
O (Outcomes)	‘relationship between the birthplace effect and the impact on talent development in soccer’
S (Study design)	‘any type of design’

**Table 2:** Questions from Downs and Black’s modified checklist used to assess the methodological quality of the included articles.

Study												
	1	2	3	4	5	6	7	8	9	10	11	TOTAL
MacDonald et al. (2009b)	1	1	0	1	1	1	0	1	0	1	1	8
Baker et al. (2014)	1	1	0	1	1	1	0	1	0	1	1	8
Lidor et al. (2014)	1	1	0	1	1	1	0	1	0	1	1	8
Ishigami (2016)	1	1	0	1	1	1	0	1	0	1	1	8
Rossing et al. (2016)	1	1	0	1	1	1	0	1	0	1	1	8
Finnegan et al. (2017)	1	1	0	1	1	1	1	1	0	1	1	9
Rossing et al. (2018)	1	1	0	1	1	1	1	1	0	1	1	9
Carvalho et al. (2018)	1	1	0	1	1	1	1	1	0	1	1	9
Nieuwstadtet al. (2021)	1	1	0	1	1	1	1	1	0	1	1	9
Smith and Weir (2020)	1	1	0	1	1	1	1	1	0	1	1	9
Teoldo and Cardoso (2021)	1	1	0	1	1	1	1	1	0	1	1	9
Maayan et al. (2022)	1	1	0	1	1	1	1	1	0	1	1	9
Brox and Krieger (2022)	1	1	0	1	1	1	1	1	0	1	1	9
Morganti et al. (2023)	1	1	0	1	1	1	1	1	1	0	1	8

1 = yes; 0 = not to determine

A structured search was carried out in relevant databases, which included PubMed (Medline), Web of Sciencie (WoS), Scopus, and SPORTDiscus. Related articles were selected from those published from 2000 to June 30, 2023.

Keywords were collected through expert opinions, scientific literature reviews, and controlled vocabulary. Search terms included “birthplace effect and soccer”, along with the Boolean operators “and” and “or”, resulting in the following unique search equation (“birthplace” [MeSH Terms] OR “birthplace” [All Fields] OR “birth of place” OR [All Fields]) AND (“soccer” [MeSH Terms] OR “soccer player” [MeSH Terms] OR “soccer” OR “soccer player” [All Fields]). Using this equation, all relevant articles were obtained. The reference sections of all the identified articles were also examined by applying the “snowball” strategy, which involves examining the reference sections of the identified articles for additional content of relevance (Sumathipala et al., 2010). No other terms were used to explore the related literature. No filters were applied regarding gender or age of athletes to increase the power of the review. No additional terms were used to increase the power of the search and analysis.

For the data collection process, the methodology consisted of reading and analysing each of the articles selected by this review’s authors. All titles and abstracts from the search were collated to identify duplicates and possible omitted studies. Titles and abstracts were then selected for a full-text review. The search for published studies was conducted independently by two authors (L.H.-S. and M.A.C.), and any disagreement was resolved by discussion with experts from third groups.

### Selection of Studies

The lead author screened the databases and selected all the studies. Two co-authors assisted with the eligibility evaluation (J.C.-G. and A.L.C.). No disagreement was found concerning the appropriateness of each of the pre-selected articles.

### Inclusion and Exclusion Criteria

The studies included in this systematic review met the following inclusion criteria: (1) studies published in peer-reviewed journals and available in full text in the cited databases; (2) studies including soccer players; (3) study designs including different countries; (4) studies including any type of soccer players, irrespective of the category; (5) studies that presented objective data on the influence of the effect of the birthplace on the development of soccer talent; and (6) studies that were published in English or Spanish in journals with a JCR (Journal Citation Reports) or a SJR (Scimago Journal Rank) impact index.

Regarding the exclusion criteria, articles analysing only collective and individual sports outside of soccer were not considered, and duplicate articles were eliminated. Furthermore, abstracts, non-peer-reviewed articles, book chapters, and conference abstracts were also excluded. Scientific articles in which the population presented some kind of disability or pathology were excluded, along with previous reviews on this topic.

Finally, two authors (L.H.-S. and J.C.-G.) independently extracted the results of the interventions using a spreadsheet (Microsoft Inc.^®^, Seattle, WA)

### Data Extraction and Analysis

Once the inclusion and exclusion criteria were applied, the information obtained was evaluated. The year of publication, the name of the journal, the authors, the sample size, the type of study, the variables selected, and the conclusions of each study were considered.

### Risk Assessment of Bias

The risk Assessment of bias was not required, as no participants were involved in the study (Manterola and Otzen, 2015).

## Results

### Search Results and Selection of Studies

**Figure 1:**
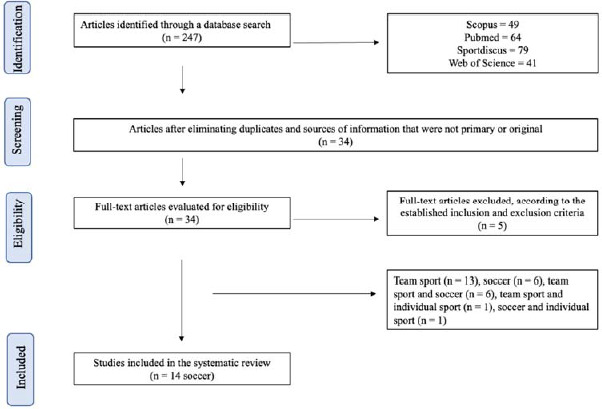
Flowchart on the literature search of the birthplace effect in soccer.

The initial literature search in the four chosen databases (PubMed, Web of Science, Scopus, and SPORT Discus) detected 247 articles on the birthplace effects and soccer. However, 213 were excluded because they were duplicates, were found to be unrelated to the birthplace effects, or did not meet the inclusion criteria (Figure 1). Of the 34 selected articles, five were excluded because they did not meet the inclusion criteria. After analysing the articles that included soccer along with other sports (individual or team), the final review of research on the birthplace effects and soccer consisted of 14 articles (Baker et al., 2014; Brox and Krieger, 2022; De Carvalho et al., 2018; Finnegan et al., 2017; Ishigami, 2016; Lidor et al., 2014; MacDonald et al., 2009b; Maayan et al., 2022; Morganti et al., 2023; Nieuwstadt et al., 2021; Rossing et al., 2016, 2018; Smith and Weir, 2020; Teoldo and Cardoso, 2021).

Table 3 (a and b) presents a summary of the most relevant information from each study in a format that is easy to understand. This table helped the authors analyse, categorise, and classify all the data necessary to carry out this systematic review.

**Table 3a:** Summary of the articles considered in the review.

Author	Title	AIM	Main Topic	Site	N Level	Sport	Dependent Variable	Outcome
MacDonald et al. (2009b)	Birthplace effects on the development of female athletic talent	To assess the influence of the BPE on the likelihood of playing a professional sport.	BPE	EEUU	188 female (76 soccer) professional athletes	Soccer Golf	City size	Female soccer players born in cities of less than 1,000,000 are overrepresented.
Baker et al. (2014)	Circumstantial development and athletic excellence: The role of date of birth and birthplace	To determine whether the RAE and BPE affect the likelihood of becoming an Olympic athlete.	BPE RAE	EEUU Canada UK German	610 male (235 soccer) Olympic athletes	Volleyball Basketball Handball Soccer	City size	Athletes born in larger cities have an advantage in selection over those born in smaller cities.
Lidor et al. (2014)	Relative age effect and birthplace effect in Division 1 female ballgame players—The relevance of sport-specific factors	To assess the influence of the BPE on the likelihood of playing a professional sport.	BPE RAE	Israel	389 female (160 soccer) professional athletes	Baseball Basketball Hockey Soccer	City size	The probability of playing in the top division was similar for players born in small and big cities.
Ishigami (2016)	Relative age and birthplace effect in Japanese professional sports: A quantitative evaluation using a Bayesian hierarchical Poisson model	To examine the influence of the BPE and its comparison with the RAE.	BPE RAE	Japan	1,824 male (1013 soccer) professional athletes	BaseballSoccer	City size Climatic conditions	The BEP influences the likelihood of professional athletes, but not because of population size, but because of climatic conditions.
Rossing et al. (2016)	The role of community in the development of elite handball and soccerlayers in Denmark	To investigate the effect of the place of early development of elite players.	BPE	Denmark	173,511 males (147,221 soccer) professional athletes	Handball Soccer	City size Density	Players are more likely to be born in high density communities. 50,000 > players < 10,000 elite athletes.
Finnegan et al.(2017)	The influence of date and place of birth on youth player selection to a National Football Association elite development programme	To determine how the RAE and BPE influence the selection of players.	BPE RAE	Ireland	1,936 male selections of future talent	Soccer	Location of elite training centres	There were unequal opportunities for access to development pathways based on the place of birth.
Rossing et al. (2018)	Influence of population size, density, and proximity to talent clubs on the likelihood of becoming elite youth athlete	To examine whether the BPE and proximity to talent clubs influence talent development.	BPE	Denmark	173,511 male (147,221 soccer) professional athletes	Soccer Handball	Population size, density, and proximity to talent clubs	High density players were overrepresented. Proximity to clubs is an important indicator.
Carvalho et al. (2018)	The birthplace of soccer players of Brazilian national teams in world cups	To investigate the effect of the place of early development of elite players.	BPE	Brazil	312 male professional athletes	Soccer	Birthplace	Distribution is heterogeneous in terms of the players’ place of birth.

**Table 3b:** Summary of the articles considered in the review.

Author	Title	AIM	Main Topic	Site	N Level	Sport	Dependent Variable	Outcome
Nieuwstadtet al. (2021)	Mechanisms explaining the birthplace effect for male elite soccerlayers	To examine the BPE not only in terms of urbanity but also in terms of the characteristics of the population.	BPE	Holland	825 male professional athletes	Soccer	Demographic rates, immigrants, and an average household income	Increased density increases the likelihood of achieving professional status in soccer.
Smith and Weir (2020)	Female youth soccer participation and continued engagement: Associations with community size, community density, and relative age	To examine participation in soccer in relation to the size of the community.	BPE RAE	Canada	9,915 female sports participants	Soccer	City size	More players participate in smaller urban settings.
Teoldo and Cardoso (2021)	Talent map: How demographic rate, human development index, and birthdate can be decisive for the identification and development of soccer players in Brazil	To examine how demographic rates and the HDI of cities can influence identification and development processes.	BPE RAE	Brazil	5,359 male professional athletes	Soccer	Demographic rates and the human development index (IHD)	There is greater potential in small- and medium-sized cities in talent identification and development processes.
Maayan et al. (2022)	The birthplace effect in 14–18 year-old athletes participating in competitive individual and team sports	To investigate the BPE of 14–18-year-old athletes and how coaches perceive this effect.	BPE	Israel	1,007 male and 390 female sports participants	Basketball Soccer Handball Volley Water polo	City size	Athletes who were born in small or medium-sized cities were more likely to reach the highest performance level in their sports.
Brox and Krieger (2022)	Birthplace diversity and team performance	To examine the influence on players’ performance according to their BPE diversity.	BPE	German	3,266 players	Soccer	Characteristics Demographics	Birthplace diversity boosts team performance.
Morganti et al. (2023)	All roads lead to Rome? Exploring birthplace effects and the “southern question” in Italian soccer	To examine the distribution of Italian soccer players.	BPE	Italy	2,012 sports participants	Soccer	Birthplace	There is an overrepresentation of players born in northern and central Italy.

BPE = Birthplace effect; RAE = Relative age effect

### Methodological Quality: Assessing the Quality of Studies

The quality ratings for each article are presented in Table 3 (a and b). The methodological quality scores ranged from 8 to 9 out of 11 points. No articles were excluded based on their methodological quality.

The Downs and Black evaluation form (Rebelo et al., 2023) contains 27 items for assessing the power of studies. The following items were selected and adjusted for this review: Is the hypothesis/purpose/objective of the study clearly described? (Item 1); Are the main outcomes to be measured clearly described in the introduction or methods section? (Item 2); Are the characteristics of the subjects included in the study clearly described? (Item 3); Are the main findings of the study clearly described? (Item 4); Does the study provide estimates of the random variability of the data for the main outcomes? (Item 5); Have the actual probability values (e.g., 0.035 instead of < 0.05) been reported for the main results, except where the probability value is less than 0.001? (Item 6); Were the participants who were asked to participate in the study representative of the entire population from which they were recruited? (Item 7); Were the statistical tests used to assess the main results adequate? (Item 8); If any of the results of the study were based on data dredging, was this made clear? (Item 9); Were the statistical tests used to assess the main outcomes appropriate (i.e., valid and reliable)? (Item 10); Were the main outcome measures accurate (valid and reliable)? (Item 11).

### Summary of the General Characteristics of the Included Articles

For publication dates, two articles were published in 2014, two in 2015, one in 2016, one in 2017, two in 2018, two in 2020, one in 2021, two in 2022, and one in 2023; these dates reflect the historical interest in the topic, which has increased in recent years. Regarding the dependent variables studied, in addition to the birthplace, six studies addressed the contextual variable of the athletes’ date of birth. Finally, in terms of the location, the studies were conducted across the globe: nine studies were performed in the Americas, four in Europe, and only one in Asia. Regarding the performance level of participants in the considered studies, it is worth noting that all of them focused on professional athletes, except for three articles focusing on sports participation; only one study focused on the selection of future talent. Studies conducted directly on soccer players included a total of 173,727 players (18,235 males and 10,492 females). Therefore, there was a clear bias in the studies towards the inclusion of male athletes. For the analysis of the effect, the filtering of articles related to soccer and both group and individual sports was analysed. From the final selection of 14 articles, six investigated the effect together with other team sports (i.e., volleyball, basketball, handball, baseball, hockey, and water polo). Only one article referred to soccer and an individual sport (golf), and the remaining seven referred exclusively to soccer.

The studies presented a variety of conclusions. Ten articles confirmed the existence of the influence of the birthplace on the development of soccer players. Four confirmed that it is more advisable to be born in cities with a larger population, while three recommended smaller cities. Regarding talent selection processes, two studies highlighted that the larger the population, the higher the chances of being selected. In terms of participation, two articles described sports participation in soccer in less densely populated cities.

## Discussion

The main objective of this systematic review was to compile the scientific literature on the effect of the birthplace on soccer performance. To the authors’ knowledge, this is the first systematic review to investigate how this contextual factor is related to talent development in soccer. As a result of the review, 33 articles were selected for inclusion and data extraction, 29 of which demonstrated a relationship between the birthplace effect and soccer performance. Using PRISMA methodology (Page et al., 2021), the analysis of the 14 articles that formed part of the final review concluded with three main discursive themes: the birthplace and population size, birthplace composition and socio-demographic characteristics of the population, and the birthplace and the location of high-performance sports centres.

### Birthplace and Population Size

Among the reviewed papers, there seemed to be some unanimity regarding the role of population size in predicting the extent to which the birthplace determines one’s professional status in soccer (Baker et al., 2014; Lidor et al., 2014; MacDonald et al., 2009b; Rossing et al., 2016, 2018; Smith and Weir, 2020). However, there was a halo of suspicion about the role of population size in determining the achievement of a professional debut based on two fundamental factors, i.e., the different territories of the studies and the sport modalities analysed.

Authors such as McDonald et al. (2009b) established that cities with less than 250,000 and 1,000,000 inhabitants offered more favourable conditions for the development of sports skills than larger cities. It is difficult to find unanimity on the criterion of the ideal size for soccer players to become professional; in fact, it was considered necessary to resort to other sports modalities to determine what the exact figure would be. The inconsistency of this variable was observed in the studies reviewed, such as that of Côté et al. (2006) or more recent studies (Maayan et al., 2022; Smith and Weir, 2020), which pointed out that smaller population areas offered more propitious conditions for experiential development than large urban environments (King et al., 2009; MacDonald, 2009b), thereby introducing the differentiation between urban and rural spaces as an additional factor worth studying.

The effect of the size of one’s birthplace varied among sports. Lidor et al. (2014) found that growing up in medium-sized cities appeared to be beneficial for female basketball and handball players, while growing up in very small places was advantageous for female volleyball players. However, no consistent advantage of the birthplace or population size was found for female soccer players.

Focusing on elite soccer players, Rossing et al. (2016) showed that they were more likely to come from densely populated communities, while handball players tended to come from smaller communities, thereby introducing the variable of density as an element to consider in the relationship between the birthplace and the size of the territory under study. For this reason, population density (inhabitants/km^2^) has been used as a key indicator when discussing the effect of the birthplace (Smith and Weir, 2020). De Carvalho et al. (2018) also focused exclusively on soccer and showed a representative heterogeneity of elite players within a territory in terms of the size of their cities, revealing predominance of players from the southeast and southern regions of Brazil.

In short, this review shows that there are several elements underlying the relationship between the birthplace and the size of a territory, one of which is the population density variable and the other being the level of equipment that a territory offers when there are certain assurances of particular population concentration. Thus, authors such as Corr et al. (2023) have argued that the physical environment of smaller cities is more conducive to deliberate leisure activities among young people of different ages and to experiment with various forms of sporting activities (Yin et al., 2022). Greater investment in infrastructure and soccer development in these areas, in addition to their significant soccer roots, suggests that fairer planning and greater investment in all regions could enhance the discovery and development of soccer talent across a country, thus underlining the importance of sports development policies that embrace and benefit all regions.

### Birthplace, Composition, and Socio-Demographic Characteristics of the Population

The inconsistency in the determination of population size could be explained by the different territorial units of analysis used in the studies, since both the city and the municipality were used in geographical contexts with varying population sizes. Overall, though, population density was found to increase the probability of achieving professional status in soccer, and this was explained by population composition, cultural diversity, and average household income (Maayan et al., 2022).

For Nieuwstadt et al. (2021), the demographic composition and cultural diversity of a population were found to be key predictors of the development of young soccer talent. Specifically, they found that participation in soccer, household income, and the percentage of non-Western immigrants predicted the number of young people who would reach elite levels in a municipality. In contrast, coaching and education resources alone did not prove to be determinants of players’ trajectories to high-performance soccer, suggesting that other factors, such as cultural diversity and playing opportunities, may be more critical for talent development (Stambulova et al., 2020).

The change from one place to another and cultural diversity within a community were found to play a relevant role (Ryba et al., 2016) in cultivating sport performance. For instance, studies conducted in the German Bundesliga (Brox and Krieger, 2022) analysed how the birthplace diversity affected team performance, with the results showing that birth diversity had an effect on team performance. Teams with a high level of birthplace diversity tended to perform better, indicating that a balanced mix of players from different backgrounds can maximise team effectiveness. This finding underlines the importance of cultural diversity and inclusion in professional sports (Stambulova et al., 2020).

Other authors, such as Morganti et al. (2023), analysed the geographical distribution of players, in this case in Italian national teams, highlighting an unequal representation, with predominance of players born in Northern and Central Italy. This imbalance suggests that regional factors, such as access to sports facilities and socio-economic differences, may significantly influence the development and detection of talent in soccer. Other studies highlighted the human development index (HDI) as a key factor (Teoldo and Cardoso, 2021) or at least a relevant indicator when referring to socio-economic issues.

Some authors have even claimed that the development of sport in a region would make people healthier, leading to better achievement of national goals in the field of sport (Yamguchi and Quetelet, 2020). Scientific knowledge generally shows that since the HDI is an indicator that considers health, education, and income variables, it somehow reflects aspects related to the quality of life of a certain region. Therefore, if a sport receives proper recognition as an indicator in the HDI, sport could play a key role in promoting public health and improving the overall quality of life (Som, 2023).

### Birthplace and the Location of High-Performance Sports Centres

In the area of the sports infrastructure, availability and accessibility for young soccer athletes stand out as relevant indicators within the players’ birthplace. These variables included training facilities, competition venues, parks, and green spaces available for unstructured play (Rossing et al., 2018; Smith and Weir, 2020).

A recent study of youth women’s soccer, in addition to analysing factors such as community size, density, and age of players, demonstrated the importance of the infrastructure and facilities by showing how census (municipal) subdivisions are linked to the use of recreational facilities, thereby suggesting an indirect role of this infrastructure in sport development (Smith and Weir, 2020). In the same vein, a study conducted in Ireland by Finnegan et al. (2017) addressed both the date and the birthplace in the search for young talent, showing that players from counties with a talent development centre were significantly more likely to be selected. Both studies (Finnegan et al., 2017; Smith and Weir, 2020), along with others, such as the work of Rossing et al. (2018), reinforce the idea that an uneven distribution of access to development programmes in territories with a nearby talent development centre could explain differences in talent selection. Thus, the main conclusion of this systematic review with respect to the infrastructure is that it is not necessary for training and competition venues to be state-of-the-art (as it is often the case in large cities); rather, venues simply need to be close by and available for frequent use. It is not just the place as a space, but what that space offers for the player’s soccer skills to develop. Therefore, we suggest that future research should focus on examining specific aspects of the general infrastructure and its correlation with sports development and performance.

### Strengths

The studies we analysed were particularly strong in terms of their samples, as they included a significant number of players within the communities in which the studies were implemented. Despite the need for further research in certain places (mainly those that produce exceptional players), the studies covered most of the world’s geography. Finally, the studies also analysed both male and female athletes.

### Limitations

Defining the birthplace is not a straightforward task. The use of postcodes, geocoding, and census subdivisions to code location and community characteristics can be an important limitation within studies addressing the effect of the birthplace. Furthermore, Hancock et al. (2022) have suggested that the birthplace does not always coincide with the place of the athlete’s development. In fact, the place of an athlete’s first club team might be a more appropriate indicator. This limitation implies that the selection of the player may need to consider both the birthplace and places in which the player developed, since these two do not always coincide. Other possible limitations include the territorial subdivisions that were made, which led to difficulties in comparing the same variables (size and density) among the countries in which the studies were carried out.

### Future Research Areas

As many of the studies pointed out, within the processes of talent development, the effect of the birthplace is a factor of great importance, and more precise research in this area is required. While the incorporation of new study variables is valuable, the interaction between these different aspects (e.g., population density, facilities, and HDI) is needed to uncover how they affect the development of a soccer player. We know that the processes of searching for talented people who can become elite are becoming increasingly refined, and paying attention to other factors (such as socio-economic, cultural, and contextual variables) is important. Research into the stimulation of players’ self-regulated learning from participation, as in street soccer, may be effective in linking the study of the birthplace effects to considerations of the type of practice undertaken by the player. However, studies are often limited to longitudinal analyses of participation trends and do not explore the underlying mechanisms that contribute to high or low participation. To the best of the authors’ knowledge, this phenomenon has not been analysed in the context of professional clubs. Taking talent processes as a foundation, future research should explore the selection and performance of young soccer players. It would also be valuable to analyse this variable with different populations to make a real and adjusted comparison between various places.

## Practical Implications

First, this review highlights the need to use more sophisticated methods to explore the birthplace effects, alongside the use of qualitative methods, to understand why some places are successful in developing talent while others are not. In addition, this study highlights the need to further explore the inclusion of a new conceptual nuance called “the place of development” in studying the birthplace effect, which has not been explored by any of the studies in the systematic review. This review is intended to be a significant milestone in building a broader and more detailed knowledge base in the field of sport talent development.

Second, the findings of this review have implications for the current practice of talent identification and development in soccer. The findings not only highlight the characteristics of the areas identified as talent hotspots, but also provide an opportunity to critically reflect on current practices of talent identification and development in soccer. There is a tendency to favour those already positioned in locations with a track record of success, thus perpetuating inequality in access to development opportunities, even within the same country. This knowledge should challenge coaches, sports managers, and policy makers to reconsider the allocation of resources. Instead of concentrating more funds on already successful areas, which could exacerbate existing disparities, decision makers could adopt a more equitable approach. This would involve investing in the infrastructure and talent development programmes in areas that have historically been neglected, with the aim of broadening the potential talent pool and promoting equity in sports in general and soccer in particular. Scouts could also benefit from adjusting their scouting strategies, thereby recognising that talent can emerge from any environment, and that a greater diversity of backgrounds could enrich the competitiveness and dynamism of professional soccer.

## Conclusions

This systematic review of the most recent scientific literature shows that the effect of the birthplace is a relevant issue in the processes of identifying, developing, and promoting talent; it thus has great importance in research into the competitive environment of team sports in general and soccer in particular.
